# Considering local immunity for innovative immunomodulatory approaches: pulmonary sepsis as a use case

**DOI:** 10.3389/fimmu.2025.1627313

**Published:** 2025-08-07

**Authors:** Emilie Vernay, Elisabeth Cerrato, François Santinon, Céline Monard, Pauline Perez, Florence Allantaz, Anne-Claire Lukaszewicz, Jean-François Llitjos

**Affiliations:** ^1^ EA 7426 “Pathophysiology of Injury-Induced Immunosuppression”, Joint Research Unit Université Claude Bernard Lyon 1 - Hospices Civils de Lyon - bioMérieux, Lyon, France; ^2^ Innovation and Partnership (I&P), bioMérieux S.A., Marcy L’Etoile, France; ^3^ Anaesthesia and Critical Care Medicine Department, Hospices Civils de Lyon, Edouard Herriot Hospital, Lyon, France

**Keywords:** sepsis, compartmentalisation, alveolar macrophages (AMs), lung infection, pulmonary microbiota

## Abstract

Owing to faster identification of sepsis and improvement of patient management, most septic patients now survive the early phase of sepsis. Therefore, one of the major challenges in sepsis management today is to identify those patients at risk and propose effective personalized therapy. The complexity of the mechanisms involved in the septic immune response and its dysregulation is reflected in the diversity of immune profiles among sepsis patients. It is now well recognized that this heterogeneity is a major obstacle to stratifying patients based on their susceptibility to secondary infections. Since sepsis can originate from different anatomical sites, some studies have investigated their impact to decipher the heterogeneity. They concluded that the site of infection affects patient outcomes and leads to different immune alterations. This narrative review focuses on pulmonary sepsis to highlight the importance of studying organ response directly with local immune cells. Understanding the persistent dysregulation within the lung, whether it involves pulmonary immune cells or other lung components, is critical. Some studies have already examined the remodeling and loss of functionality of alveolar macrophages after the initial insult. Ongoing research is also investigating the impact of imbalances in other lung players, such as epithelial cells or the microbiota, on susceptibility to pulmonary reinfection.

## 1 Introduction

Sepsis is defined as a dysregulated host response to infection, resulting in life-threatening organ dysfunction (1). In 2017, sepsis affected around 49 million people and was responsible for approximately 11 million deaths worldwide (2). In addition, sepsis is associated with significant long-term health issues, including higher rates of mortality after discharge, and physical and cognitive impairments (3). Due to its high incidence rate and associated complications, sepsis represents a major health concern (4).

Over the past decades, numerous studies have aimed to elucidate the pathophysiology of sepsis, with most data coming from the circulating compartment. Initially, the sepsis model was thought to be biphasic, starting with an initial phase of hyperinflammation including the secretion of pro-inflammatory cytokines and neutrophil recruitment (5). In this model, this pro-inflammatory phase was balanced by an immunosuppressive compensatory phase to restore homeostasis. The hallmarks of this anti-inflammatory phase comprise a decreased monocytic HLA-DR expression, T cell exhaustion and lymphocyte apoptosis (6). However, recent studies have challenged this model by identifying immunosuppressive features during the early phase of sepsis. Thus, the current consensus is that pro-inflammatory and anti-inflammatory processes occur simultaneously (7), with the early phase dominated by hyperinflammation and, in cases of unresolved sepsis, followed by a persistent immunosuppression (8).

Advances in understanding the early phase of sepsis and improved patient management have reduced the mortality of sepsis in the first 48 hours. However, patients remain at high risk of secondary infections, which have a serious impact on their short and medium-term prognosis (9). A retrospective study estimated that approximately one third of septic patients develop a nosocomial infection during their ICU stay, leading to an increased risk of mortality and prolonged hospitalization (10). Over the past years, a growing amount of clinical and experimental data has shown that this susceptibility to secondary infections is associated with persistent immunosuppression resulting from an imbalance in the host response (11).

Identifying patients at higher risk of secondary infections remains a challenge. Although several diagnostic solutions have been proposed, their performance does not provide a sufficient level of accuracy and does not allow their use in clinical routine (12–14). One of the reasons limiting their use is the wide range of responses that are observed, due to the heterogeneity of immune profile following initial inflammatory response. Several reasons have been suggested to explain this heterogeneity, such as host-related factors, nature or site of initial infection (15). Interestingly, data examining the host response in patients according to the source of sepsis suggest the existence of organ-specific immune alterations (16). Although scarcely evaluated to date, the heterogeneity of the immune response at the organ level, as well as the source of infection, represents an opportunity to advance our understanding of the mechanisms underlying sepsis. It may also improve the accuracy and relevance of diagnostic tools to identify patients at risk of secondary infection.

The aim of this review is to discuss the key role of local immune alterations in sepsis and to provide new perspectives for the development of immunomodulatory therapies to prevent secondary infections.

## 2 Heterogeneity of sepsis population

Although it is now well-accepted that septic patients are highly heterogeneous, the underlying mechanisms of this variability remain poorly understood. The sources of heterogeneity in the pathophysiology of sepsis are diverse and include the pathogens causing the infection (17), organ dysfunction, host responses, host factors and the primary site of infection. Hereafter, we discuss how the origin of the infection leading to sepsis influences outcomes and the organ-specific immune response.

### 2.1 Source of sepsis impacts clinical outcome and systemic host response

Sepsis is a syndrome defined by an uncontrolled immune response to infection, resulting in organ dysfunction. It can originate from a variety of anatomical sites, including the abdomen, lungs, central nervous system, skin and soft tissues, and genitourinary system. This definition suggests that the systemic immune response remains consistent, irrespective of the location of the infection.

Nevertheless, comparative analyses of patient outcomes across sepsis cohorts have demonstrated that clinical trajectories (particularly organ failure and length of stay) vary according to the initial source of infection (18). These differences were observed even when initial severity was similar between groups (19). In their retrospective study, Leligdowicz et al. (20), examined the association between initial site of infection and hospital mortality among sepsis patients, adjusting for predisposing factors such as comorbidities. Their findings suggested that there were differences in hospital mortality according to the initial site of infection. Specifically, intra-abdominal infections, lower respiratory tract infections, and biliary tract infections were associated with higher mortality rates. A similar conclusion was also drawn in a large cohort of sepsis patients in the UK (21). Furthermore, a longitudinal study demonstrated that the source of the initial infection in sepsis patients affects not only in-hospital mortality but also long-term outcomes (22).

Apart from mortality, anatomical sources of infection also influence the susceptibility to secondary infections. In a study focused on pulmonary sepsis, Llitjos et al. observed that an initial pulmonary infection increased susceptibility to a subsequent pulmonary infection (23). Similar results were also observed in another study (24).

To explore the systemic immune response, studies have compared several blood-based markers in septic patients, stratified by infection site. In the MARS cohort, transcriptomic analyses of peripheral blood revealed both shared and site-specific immune signatures (16). Furthermore, the levels of plasma cytokines involved in inflammation, endothelial cell activation, and coagulation activation vary according to the origin of sepsis. Stortz et al. corroborated these findings, demonstrating discrepancies in the plasma levels of pro-inflammatory markers among septic patients categorized by the initial infection site (22). Differences at cellular levels were also reported. A longitudinal evaluation of the absolute lymphocyte count (ALC) was conducted during the initial 14 days of intensive care unit (ICU) treatment. It revealed distinct recovery trajectories to normal ALC ranges among the study’s subgroups.

Taken together, these data demonstrate that different systemic immune responses are elicited depending on the location of the initial infection. A comprehensive analysis of sepsis, informed by the anatomical location of the infection, holds potential to elucidate the heterogeneity in immune responses and enhance the precision of clinical predictions, including mortality and length of hospital stay. Additionally, given that the primary sources of sepsis influence susceptibility to secondary infections, this suggests distinct biological immune alterations across organs.

### 2.2 Existence of a multilevel organ specific immune response

Previously cited studies have focused on the analysis of blood samples to investigate how the host immune response varies with the primary site of infection in sepsis. These differences may be attributed to the presence of organ-specific immune mechanisms. Murine models are frequently used to investigate these responses in depth due to the ease with which immune responses in different tissues can be evaluated. The most common murine models of sepsis involve two distinct approaches: cecal ligation and puncture (CLP), which mimics abdominal sepsis, and injection of lipopolysaccharide (LPS).

These models have enabled comparisons of transcriptomic dysregulation across organs (25–27). In particular, following sublethal injection of LPS, transcriptomic mapping of 13 different tissues unveiled organ-specific signatures of sepsis (26). The longitudinal assessments of gene expression in these tissues revealed different recovery capacities among organs. Similar results were obtained in a CLP model, where many dysregulated genes implicated in inflammatory responses, including interleukin-1β (IL-1β), reactive oxygen species (ROS) generation, and complement proteins, were differentially expressed across organs (27). These results were corroborated at the protein level. For instance, the production of tumor-necrosis factor alpha (TNF-α) in the peritoneum was found to be 16 times higher than in the blood using a CLP-induced sepsis model. While TNF-α levels normalized in the peritoneum within 24 hours, they remained elevated in the lungs for 5 days. This highlights differences in both the magnitude and duration of the inflammatory response between organs (28).

Beyond inflammatory mediators, organ-specific immune responses also involve other molecular pathways. In mice exposed to systemic LPS, lung and kidney tissues showed different expression levels of adhesion molecules and proteins involved in maintaining endothelial integrity (29). Since leukocyte recruitment to infected organs depends on their ability to cross the endothelial barrier, these findings demonstrate that organ-specific differences in endothelial function can influence immune cell infiltration.

Indeed following CLP, quantitative and functional variations in tissue macrophages are observed, with the magnitude and direction of these changes varying according to the tissues examined (30). Similarly, the intravenous injection of LPS into humans has been demonstrated to elicit a shift in the responsiveness of alveolar macrophages (AMs) *in vivo* (31). Within the first 6 hours, AMs exhibited enhanced functional responsiveness to secondary *in vitro* stimulation. Nonresident cells also undergo modifications in an organ-specific manner following systemic inflammation. A study of the response of natural killer (NK) cells in mice injected with endotoxin revealed differences in recruitment and activation across the lungs, spleen, and blood (32). Through adoptive transfer, Rasid et al. demonstrated that NK cells display organ-specific responses influenced not only by their tissue of origin but also by local remodeling within the organ microenvironment.

These data underscore organ-specific immune responses that vary in intensity and modify tissue-resident cell responsiveness. As different infectious sites elicit distinct immune responses, the organ of origin may undergo specific modulation following initial injury. Some human studies have investigated the impact of infection site on local inflammation. In experimental endotoxemia, comparing endobronchial to intravenous LPS administration, alveolar cytokine levels were measured in the hours following the challenge (33). Bronchoalveolar lavage from volunteers who received pulmonary LPS instillation showed higher levels of proinflammatory cytokines than those who received intravenous administration, confirming that the kinetics of the immune response depend on whether the organ is the primary site of infection. As the site of infection undergoes a rapid and intense inflammatory response, it may disrupt local immune balance. Indeed, AMs, from patients with pulmonary sepsis exhibited reduced HLA-DR expression in comparison to those with abdominal sepsis (34). As low HLA-DR expression is recognized as a hallmark of immunosuppression, it indicates that the site of infection undergoes suppressive modulation of its local immunity. Local immunosuppression in the days following the initial insult was confirmed in a mouse model of sepsis comparing pulmonary immune responses after abdominal or pulmonary infection (35). Comparison of murine models revealed distinct AM depletion and reduced bacterial clearance following pulmonary sepsis. This immune alteration was associated with reduced survival after a secondary pulmonary infection. In a mouse model with intravenous LPS instillation, it was confirmed that immunomodulation depends on whether the considered organ is the source of sepsis, with lung cells exhibiting lower endotoxin tolerance than circulating cells (36).

Sepsis affects organs differently, with immune responses being organ specific in terms of cellular response, transcriptomics and proteomics. In addition, the post-acute phase differs depending on whether the organ is the primary source of infection or not, with persistent modulation localized to the primary infected organ.

## 3 Compartmentalised immune response in sepsis: pneumonia as a use case

Given that organs exhibit unique immune responses, especially when they are the primary site of infection, a detailed examination of local immunity could help elucidate the heterogeneity of sepsis patients. We will focus on pneumonia as it is the most common source of community sepsis in high-income countries (21, 37, 38) and the most frequent source of healthcare-associated infections (39). Sepsis induced by pneumonia is associated with both increased mortality compared to other sites of infection and high risk of secondary infections (40). We will focus on bacterial infections, which account for the majority of severe lung infections (41).

### 3.1 Immune response to pneumonia differs between circulating and lung compartments

Most studies on sepsis focus on peripheral blood, regardless of the infection site, due to its accessibility. It is commonly assumed that the results obtained from peripheral blood are a pertinent surrogate for the local immune response. However, the diagnostic performance of circulating biomarkers is poor in both sepsis and pneumonia (42). For instance, circulating procalcitonin (PCT) and C-reactive protein (CRP) exhibit poor performance in identifying patients with positive culture among those suspected of pulmonary infection (43). Similarly, plasma levels of interleukin-6 (IL-6) and interleukin-8 (IL-8), two pro-inflammatory cytokines, were similar in mechanically ventilated patients with and without a nosocomial pneumonia (44).

**Figure 1:**
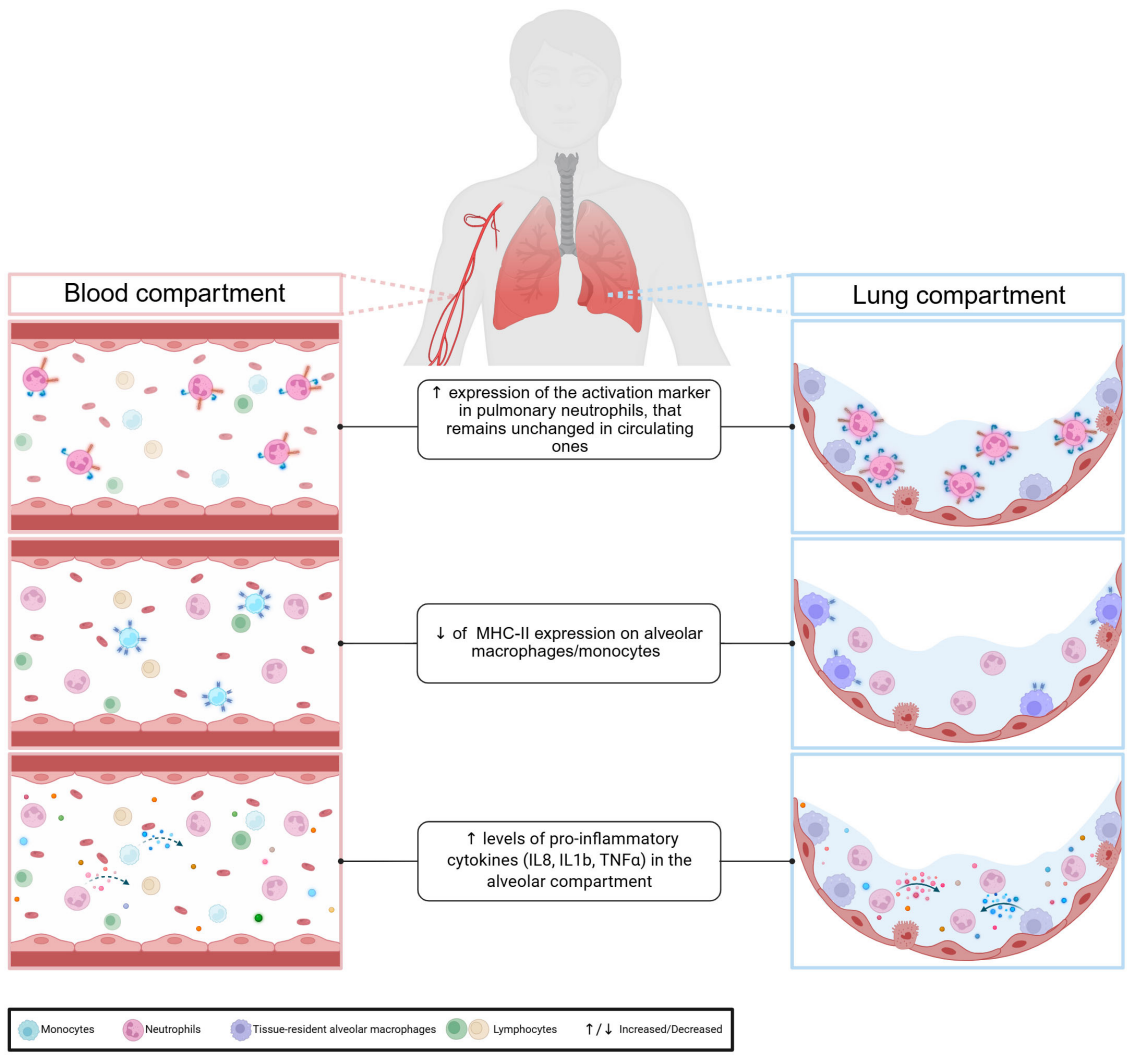
Compartmentalisation of pulmonary immune response.

Given the limitations of blood biomarkers, several studies have examined markers of the immune response in both blood and lung compartments (**Figure 1**). As the pulmonary immune response is associated with strong neutrophil infiltration in the alveolar space, the neutrophil chemoattractant IL-8 has been of particular interest (45). Conway et al. compared serum and alveolar levels of IL-8 and other pro-inflammatory cytokines in patients with and without ventilator-associated pneumonia (VAP) (46). None of the serum cytokines evaluated could discriminate between the two groups, unlike alveolar cytokines, all of which showed higher levels in infected patients. Similar results were obtained in COVID-19-related ARDS patients, with endotracheal aspirate/serum cytokines ratios ranging from approximately 2 to 30,000 (47). Bendib et al, confirmed higher levels of alveolar proinflammatory cytokines, particularly IL-8, and noted differences in cellular activation markers between blood and lung compartments (48). In pneumonia patients, TNFα concentrations in bronchoalveolar lavage fluid (BALF) were, on average, ten times higher than in blood (49). This variability is also found within the organs themselves, with a regionalization of the immune response. In patients with unilateral lobar pneumonia, the concentration of IL-6 is significantly higher in the affected lung lobe compared to the unaffected one (49).

Beyond quantitative differences, studies also reveal phenotypic and qualitative differences. In septic shock, monocytic HLA-DR expression differs significantly between blood and lung compartments (34). Another study reported that in a cohort of community acquired pneumonia, alveolar neutrophils expressed more activation markers than circulating neutrophils (50). Taken together, these data confirm that due to the compartmentalisation of the immune response, blood is a poor reflection of organ-specific immune responses. Therefore, to have a comprehensive view of the organ-specific immune response induced by pulmonary sepsis, we should focus on the immune response at the alveolar level.

### 3.2 Pulmonary inflammatory response in lower respiratory tract

#### 3.2.1 Lower respiratory tract at homeostasis

**Figure 2:**
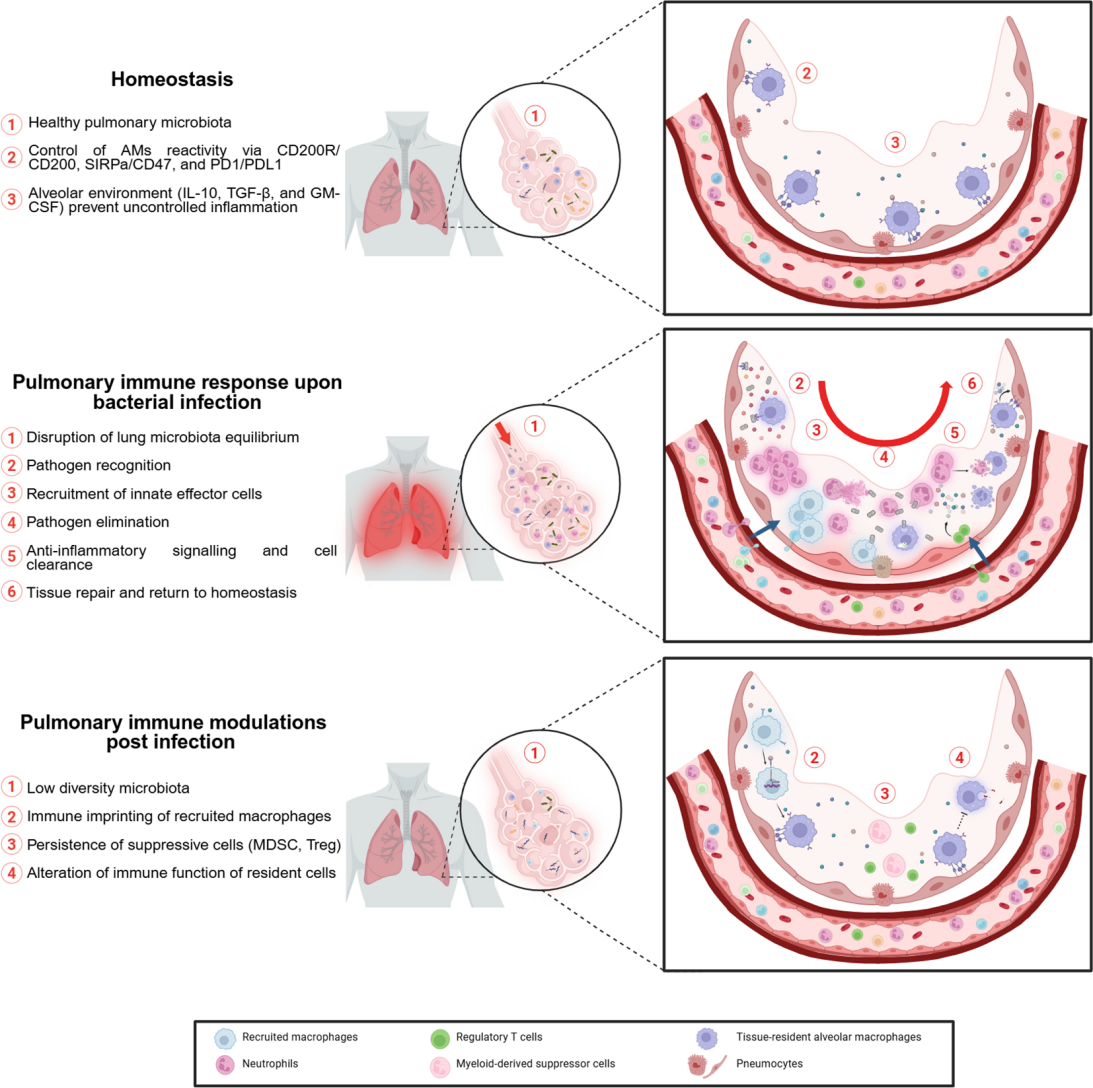
Impact of pulmonary bacterial infection on local environment.

At homeostasis the most abundant cells found in the lung are AMs. These tissue-resident cells are formed during prenatal stages (51) and act as patrolling cells to eliminate pathogens. AMs are a key component of pulmonary immunity as they are involved in inflammation but also in the return to homeostasis (52). AMs closely interact with alveolar epithelial cells (AEC), notably through cell-to-cell interactions involving CD200R, PD1, and SIRPa on macrophages and CD200, PDL1 and CD47 on alveolar epithelium, respectively (53). In addition to direct cell-to-cell interactions, AECs also secrete regulatory molecules, such as interleukin-10 (IL-10), granulocyte-macrophage colony-stimulating factor (GM-CSF), and transforming growth factor beta (TGF-β), that help maintain AMs in a steady state and prevent excessive inflammation (54, 55) (**Figure 2**). Recently, the study of the pulmonary immune response has included a less conventional player than immune cells: the microbiota. Its role in the development of respiratory infections and its interaction with the cells involved in the pulmonary immune response have been of particular interest (56, 57).

#### 3.2.2 Lung immune response to infection

The early phase of lung infection involves mainly resident AMs, which account for approximately 90% of patrolling cells in alveoli (58) and recruited neutrophils (59). Effector cells of the lung’s innate immune response eliminate bacteria through mechanisms such as phagocytosis (60), release of cytolytic molecules (e.g., reactive oxygen species, lysozyme, defensins) (61), and formation of neutrophil extracellular traps (NETs) (62). Alveolar epithelial cells also help initiate the immune response by secreting pro-inflammatory cytokines, including interleukin-33 (IL-33) (63, 64). This alarmin enhances host defense during pulmonary infection (65). Furthermore, through crosstalk, alveolar epithelial cells serve as gatekeepers for AMs responsiveness. Thus, their activation is critical for an efficient macrophage immune response (66). Innate immunity not only eliminates pathogens but also initiates the adaptive immune response. This process is mediated by antigen-presenting cells (APCs), including monocytes, macrophages, and dendritic cells (DCs). Although AMs have high phagocytic activity, their role as APCs remains unclear (67). APCs activate T cell response, which in the case of pulmonary bacterial infection is mainly mediated by Th17 cells (68). These polarized T cells secrete interleukin-17 (IL-17), which enhances the innate response, for example, by improving neutrophil recruitment (69).

To prevent tissue damage from excessive inflammation, the lung relies on a feedback loop. Resolution involves cell death of immune cells recruited during infection and their clearance to restore normal lung composition. Cell death occurs through various mechanisms, including apoptosis, necrosis, and pyroptosis (70, 71). These processes are triggered either by intracellular programming or by extracellular signals released from activated innate immune cells (72). Most mechanisms trigger the release of proteins and cellular debris identified by immune cells as damage-associated molecular patterns (DAMPs), which in turn amplify the inflammatory response. For example, when neutrophils undergo cell death, vesicles contained in their cytoplasm release cytolytic enzymes, that can cause tissue damage (73). To prevent this, phagocytic cells and particularly AMs are responsible for eliminating dying cells by efferocytosis (74). Dying cells display ‘eat-me’ signals, either soluble or membrane-bound, that help AMs recognize and clear them. For instance, through the TYRO3, AXL and MERTK (TAM) family of receptors, AMs can recognize apoptotic cells by binding to phosphatidylserine on their surface (75). Removal of dying cells by AMs induces a shift (52) from pro- to an anti-inflammatory profile promoting return to homeostasis and tissue repair. Noteworthy, murine AMs have demonstrated self-induction of immunosuppressive signaling following infection (76). To facilitate the return to homeostasis AMs secrete immunosuppressive cytokines that promote the dampening of inflammation (77). Tissue repair is promoted by AMs through activation of AECII proliferation (78), which can transdifferentiate into type I cells to rebuild the alveolar-capillary barrier (79). In addition, AMs are responsible for the homing of memory lymphocytes to the mucosa through prostanglandin E2 (PGE2) signaling, providing long-term immunity (80).

AMs are not the only cell type involved in the pulmonary feedback loop. Zhang et al, demonstrated that instillation of apoptotic cells into LPS-infected mouse lungs enhanced resolution of inflammation (81), in particular by inducing recruitment of functional lymphocyte T regulators (T reg). The role of Treg in the resolution of pulmonary inflammation is multifaceted. They are important sources of suppressive cytokines but are also capable of enhancing anti-inflammatory mechanisms such as AM efferocytosis (82). They also act as suppressors of immune function, notably by regulating IL-17 secretion by Th17 cells (83) and gamma delta (γδ) T cells (84), to prevent uncontrolled secretion that can lead to chronic disease (85). Among lymphoid cell types, unconventional lymphocytes, which include innate lymphoid cells (ILCs) and γδ T cells, also participate in the anti-inflammatory response. They favor cell clearance through cytotoxic activity (86) and support tissue repair through secretion of GM-CSF, which is involved in maintaining and replenishing the pool of AMs (87).

In summary, host defense involves a coordinated process, from pathogen recognition and elimination to restoration of homeostasis (**Figure 2**). This fragile balance between pro- and anti-inflammatory mechanisms can be disturbed by uncontrolled inflammation during sepsis. Thus, unraveling the pulmonary modulation resulting from the immune response could help explain susceptibility to lung reinfection and provide new approaches to boost immunity and prevent reinfection.

## 4 Increased susceptibility to secondary infections due to imbalanced pulmonary immune response

Similarly to the systemic response, the pulmonary immune response combines pro- and anti-inflammatory signals that are tightly regulated to provide sufficient and balanced inflammation to resolve infection without harming the host. In the context of pulmonary sepsis, immune dysfunction characterized by an uncontrolled inflammatory response is associated with tissue damage leading to lung injury or development of acute respiratory distress syndrome (ARDS) (88). Susceptibility to secondary infections is generally associated with persistent immunosuppression (89). In peripheral blood, it is characterized by uncontrolled apoptosis of immune cells, altered cell functionality and high levels of immunosuppressive factors (6). In parallel with these systemic alterations, we next examine how the pulmonary immune environment is modified following a primary infection, with particular attention to local immune dysfunction (**Table 1**).

**Table 1:** Alteration post-infection of lung environment.

Murine model	Human data
Modification of cellular composition	
• Depletion of resident AMs replaced by monocyte-derived macrophage • Recruitment of immunosuppressive cells (MDSC, Treg)	• Identification of distinct AMs subsets
Functional alterations	
• ↓ Phagocytic activity of AMs • ↓ Antigen presentation by DCs	• Transcriptional hyperinflammatory AMs subsets
Modification of pulmonary microbiota	
• Dysbiosis after LPS instillation	• Dysbiosis characterized by reduced diversity • ↓ abundance of anaerobic and oral commensal communities

### 4.1 Post-infection cells remodeling

As tissue-resident cells, the maintenance and replenishment of the AMs pool during infection are critical. After infection with *S.pneumonia*, mouse AMs are depleted due to antimicrobial activity (90, 91). However, 35 days after infection, the absolute number of AMs was equivalent to the basal level. Using a mouse model of LPS pulmonary instillation, characterization of replenished AMs revealed that they were mainly newly recruited and differentiated from circulating monocytes (92), suggesting monocyte recruitment rather than proliferation.

In addition to phenotypic changes, infection also induces functional remodeling of AMs. In mice, both resident and recruited macrophages were transcriptionally activated up to 12 days after the onset of infection compared to baseline (93). However, the activated pathway and cytokine secretion differed between resident and recruited macrophages. In a similar model, 7 days after intratracheal instillation of LPS, recruited macrophages showed enrichment in pathways related to immune response and signal transduction. In contrast resident macrophages were enriched in PPAR signaling and fatty acid metabolism and degradation signaling (94). Functional assays of both type of macrophages identified that efferocytosis ability was higher in resident AMs.

To explain these functional differences, it has been proposed that the alveolar niche plays a pivotal role in regulating AMs activity through a process known as “tissue immune imprinting” (95). According to this model, resident AMs are shaped by the alveolar microenvironment, which limits their plasticity and promotes a hyporesponsive phenotype, helping to prevent excessive immune activation and facilitates rapid resolution. In contrast, during inflammatory episodes, circulating monocytes are recruited to the lung and differentiate into macrophages that retain a pro-inflammatory epigenetic program. Thus, newly recruited macrophages exhibit greater plasticity and are more prone to mounting exaggerated inflammatory responses. Therefore, alveolar niche imprinting is essential for maintaining the homeostatic function of AMs and preventing dysregulated immune responses.

While murine models of infection have identified distinct phenotypes for AMs and characterized the kinetics of each subset, little is known about this heterogeneity in humans. Recently, the heterogeneity of the AMs population at homeostasis was confirmed by single cell RNA-seq, which identified distinct subsets of AMs in BALF from healthy volunteers (96). In patients with mild or severe pneumonia, similar analyses revealed the emergence of hyperinflammatory subsets of neutrophils and AMs that were absent in healthy controls (97). Morell et al. further identified up to six transcriptionally distinct AM clusters in BALF from patients with acute hypoxemic respiratory failure, with one subset correlating with the severity of acute respiratory distress syndrome (ARDS). These studies pave the way for further functional and clinical characterization of AMs subsets, as their specific roles in pulmonary immune responses and disease progression remain to be fully elucidated.

### 4.2 Impact of alveolar remodeling on susceptibility to infections

Following primary infection, not only is the cellular composition of resident cells modified, but their functionality is also impaired. Using a mouse model of sequential pulmonary bacterial infection, Roquilly et al. investigated the functional changes induced by a first infection on alveolar cells (98). They observed reduced bacterial clearance in the lungs of mice infected twice compared to once. Evaluation of different immune functions of AMs from infected mice showed reduced phagocytic capacity without changes in major histocompatibility complex II (MHCII) expression or LPS responsiveness. Using adoptive transfer of naive AMs, the authors found that the alteration in phagocytic capacity depends on early signal regulatory protein α (SIRPα) signaling, which modulates the alveolar environment after infection. Thus, AMs paralysis results from an unbalanced immune response that perturbs the local immune environment. Consistent with these findings, another study showed that alterations in phagocytic capacity occurred only when the initial infection was pulmonary (35). Alteration of cell functionality is not limited to AMs. In a double pneumonia mouse model, it was observed that dendritic cells (DCs) developing in the lung after resolution of primary pneumonia had a reduced capacity to present antigens and secrete immunostimulatory cytokines (99). However, no DC alteration was observed in the spleen, highlighting the compartmentalisation of immune changes to infected tissues.

In another aspect, studies have reported recruitment of immunosuppressive cells associated with impaired immune response. Repeated pulmonary infection with *A.Baumannii* is associated with an increase in the Treg population in the lung altering the pro-inflammatory response (100). Myeloid-derived suppressor cells (MDSCs), suppressive cells derived from the myeloid lineage, have been reported to be recruited into the alveoli during bacterial infections (101). However, their potential beneficial or detrimental effects remain unclear. In *K.pneumoniae* infection, they were found to participate in resolving lung inflammation and have reparative function (102, 103). On the contrary, they promoted lung injury in a mouse model of severe tuberculosis (104). While suppression of T cell functions by MDSCs is well documented, their impact on AMs functions remains to be determined. For example, MDSCs induced by fungal infection impair AMs phagocytosis (105). As all these studies are based on the mouse model, the role of MDSCs in pulmonary sepsis on alveolar cells remains to be determined in humans.

This highlights the importance of murine models in providing evidence of profound modulation of pulmonary immunity following initial infection. Notably, pneumonia-induced remodeling of AMs subsets has been shown to be long-lasting, with phenotypic and transcriptional changes in murine AMs lasting up to six months after infection (106). These alterations include changes in the phenotype of resident lung cells as well as their functionality. The unbalanced immune response is also reflected by modification of the lung environment including immunosuppressive features. This underscores the importance of understanding how infection sequelae can disrupt mechanisms that maintain the balance of the pulmonary microcosm. Indeed, the existence of a pulmonary microbiota implies the presence of tolerance mechanisms towards commensal bacteria by resident pulmonary immune cells. However, the paralysis of their microbicidal mechanisms could create a favorable environment for commensal pathogenic bacteria to proliferate, leading to secondary infections.

### 4.3 The importance of the lung microbiota and its modulation

Contrary to the old belief that the lung is a sterile environment, it harbors a rich microbiota consisting of bacteria, viruses and fungi (56). The microbiota is continuously renewed, but the core lung microbiome includes genera such as *Pseudomonas*, *Streptococcus*, *Proteus*, *Clostridium*, *Haemophilus*, *Veillonella*, and *Porphyromonas.* Compared to the upper respiratory tract, the lung microbiota harbors the same bacterial populations but with a lower biomass (107). So far, the dynamics of the microbiota in pulmonary infections remain poorly characterized.

#### 4.3.1 Pulmonary infection and microbiota dysbiosis

Because mechanical ventilation provides easier access to lung samples, most studies have examined the microbiota in ventilated patients either at the time of diagnosis of pulmonary infection or through longitudinal assessment after intubation. Fenn et al. found that the microbiota in patients with ventilator-acquired pneumonia was characterized by lower diversity with a predominance of pathogenic bacteria (108). Dysbiosis of the lung microbiota was also observed in diverse clinical context of pulmonary infection (109, 110). To identify if specific communities were impacted by microbiota dysbiosis, Kitsios et al. conducted a longitudinal study of the microbiome during the first two weeks of intubation in patients with acute respiratory failure (111). They observed a reduction in the abundance of anaerobic bacteria and oral commensal bacteria. They also showed that patients who maintained low microbiome diversity over the two-week period had worse survival outcomes, linking disease severity to microbiome dynamics. All studies concluded to a shift from a “healthy,” well-balanced polymicrobial microbiome to an “unhealthy,” restricted, and less diverse microbiota. These observations were confirmed in murine models of LPS-induced lung injury (112). However, due to the timing of sampling, often at the point of infection diagnosis, and the bacterial origin of the infection, it remains unclear whether dysbiosis is a cause or a consequence of secondary pneumonia.

#### 4.3.2 Pulmonary microbiota dynamics

The dynamics of the lung results from three mechanisms: microbial immigration, microbial elimination, and the proliferation rates of its members. Dickinson et al. proposed that the microbiome in the healthy lung is determined by the balance between immigration and elimination mechanisms (113). Microbes originating from the air and upper respiratory tract migrate to the lower respiratory tract (114) and their numbers are regulated by numerous mechanisms such as coughing or mucociliary clearance. To date, the dysbiosis characteristic of pulmonary infection is thought to result from changes in endogenous growth conditions through mechanisms that are not yet fully understood, despite various hypotheses (115). Modification of the lung environment can result from abiotic or biotic factors. Abiotic factors include temperature, pressure, nutrient accessibility, but also mucus or pulmonary surfactant secretion, which are modified during the host response. It can also be modified by external interventions. For example, mechanical ventilation has been associated with a decrease in bacterial alpha diversity (116). The microbiota is also modulated by surrounding cells. As the lung is colonized with bacteria, at homeostasis, host-microbiota interactions exhibit symbiotic relationships to promote tolerance. Upon infection, lung cells are mobilized to mount an immune response, disrupting their basal state of activation.

#### 4.3.3 Host-microbiota crosstalk

The interaction between microbiota and lung cells is a two-way crosstalk. At homeostasis, microbiota diversity influences the cytokine lung environment and cell composition (117, 118). AMs from microbiome-depleted mice show impaired ROS production in response to *in vitro* infection with *K.pneumoniae* (119). Moreover inflammatory signaling was impaired in mice lacking microbiota after infection with *K.pneumoniae* or *S.pneumoniae*, in particular, with lower levels of GM-CSF and IL-17A, two cytokines participating in host defense (120). However, most studies have investigated the global impact of microbiota on the immune response, rather than the importance of individual species. One study, identified *Prevotella* as an innate response enhancer that provides protection against infection in mice through pattern recognition receptor signaling (121). Further studies are needed to determine the impact of different species depletion on host immune cells, particularly to identify mechanisms of host-microbiota crosstalk that may provide new potential targets for enhancing the immune response.

Despite growing evidence that pulmonary infection is associated with microbiota dysbiosis, host-microbe interactions are complex and bidirectional, complicating the understanding of microbiota dynamics during infection (115). To elucidate the role of dysbiosis in susceptibility to secondary infection, either by favoring the outgrowth of certain bacteria or by altering immune cell function, several limitations in current research need to be addressed. These include overcoming of technical limitations (e.g low biomass, anaerobic microorganisms), expanding sequencing libraries to include viruses and microbial eukaryotes, describing the healthy dynamics of the microbiome, and accounting for interpersonal variability and confounding by prioritizing longitudinal studies.

## 5 New approaches for immunomodulatory therapies

Sepsis is characterized by an unbalanced immune response, making immunotherapy a promising approach to regulate the immune response in patients. Despite promising results in animal models, all clinical trials have failed to demonstrate a beneficial effect of immunomodulatory therapy. The limitations and reasons for these failures have been extensively reviewed, highlighting the importance of addressing the heterogeneity of immune profiles (122–124). To address this, several avenues for future research have been proposed, all pointing towards a comprehensive description of sepsis-induced alterations. Considering the compartmentalisation of the immune response will not only help to decipher this heterogeneity but will also provide a complete picture of all sepsis-induced local modulations, including all local actors involved in maintaining homeostasis. Identification of key mechanisms favoring the onset of nosocomial infections thus provides new potential targets for novel immunomodulatory strategies. Here, we will focus on the post-infection phase of pulmonary sepsis and how the dysregulation between pro-inflammatory signaling and compensatory mechanisms could be modulated to prevent secondary infection, either by directly targeting the immune system or indirectly by modulating the microbiota (**Table 2**).

**Table 2:** Immunomodulatory strategies to improve management of pulmonary infection in ICU.

Compound	Therapy	Study population	Clinical trial	Administration	Main therapeutic objective
Cytokines-based strategies					
rhGM-CSF	Molgramostim	Patients with pneumonia-associated ARDS	GI-HOPE (NCT02595060) Phase 2	Inhaled	Enhance host defense and repair lung epithelium
	Sargramostim	Healthy volunteers & ICU patients	INSIGHT-AM (152) Phase 2	Inhaled	Improve AMs function
rhIFNg	Imukin	Mechanically ventilated ICU-patients	PREV-HAP (126) Phase 2	Subcutaneous	Prevent hospital-acquired pneumonia
		Mechanically ventilated ICU-patients	IGNORANT (NCT05843786) Phase 3	Subcutaneous	Reduce ventilation duration after VAP in immunosuppressed patients
Microbiota directed strategies					
TLR5 agonist	Flamod	Healthy volunteers	NEBUFLAG Phase 1	Inhaled	Stimulate host response to enhance effectiveness of first-line antibiotics
L. rhamnosus	L. rhamnosus GG	Mechanically ventilated ICU-patients	PROSPECT (146) Phase 4	Ingested	Prevent VAP

### 5.1 Immunotherapy to modulate locally sepsis-induced alterations

Immunomodulatory therapy aims to reverse defective immune function and boost the immune system. In the context of sepsis, several cytokines have been tested as immunomodulatory therapies, including IL-7 (125), GM-CSF and interferon-γ (IFN-γ) (126). In particular, in the context of pulmonary infection, a correlation has been found between lack of GM-CSF and IFN-γ and AMs paralysis (127). After *in vivo* LPS pulmonary instillation and depletion (or not) of alveolar GM-CSF and IFN-γ, AMs from mice were harvested and challenged with LPS *in vitro*. Surprisingly, AMs did not show endotoxin tolerance except in mice lacking alveolar GM-CSF or IFN-γ making these two cytokines particularly interesting as potent immunotherapies.

IFN-γ has pleiotropic effects on the innate and adaptive immune system. In sepsis clinical trial, recombinant IFN-γ was proved to restore HLA-DR expression on monocytes. Since reduced expression is considered a hallmark of sepsis-induced immunosuppression, this makes IFN-γ a good candidate for reversing immunosuppression in critically ill patients. However, the use of peripheral administration of recombinant IFN-γ did not reduce the incidence of hospital-acquired pneumonia among critically ill patients (126). With the growing importance of considering organ-specific immune responses, new studies are evaluating the effect of local administration. For example, prophylactic treatment with intranasal administration of recombinant IFN-γ provides protection against SARS-CoV-2 infection in a mouse model (128).

Unlike IFN-γ, which is known for its role in enhancing the immune response, GM-CSF is involved in maintaining alveolar homeostasis, recruiting myeloid cells, and differentiating newly recruited macrophages into resident AMs (129). As a result, GM-CSF is emerging as a promising therapeutic agent for modulating pulmonary immunity. Clinical trials in sepsis patients have shown that peripheral administration of recombinant GM-CSF can reverse the hyporesponsiveness of circulating monocytes (130). Local administration of GM-CSF has been shown to improve survival rates in mice with secondary pneumonia (131). In support of these findings in animal models, preliminary data from a limited number of patients have provided initial insights into the effects of this therapy on pneumonia-associated ARDS, suggesting enhanced activation of AMs (132). To further elucidate the potential beneficial effects of inhaled GM-CSF on AMs, several ongoing studies are investigating its effects in critically ill patients (ISRCTN78203402, NCT02595060).

Advances in understanding sepsis-induced changes have identified novel pathways as potential therapeutic targets. IL-33, an alarmin produced by injured tissues, has been shown to contribute to persistent immunosuppression during sepsis (133). Therefore, targeting the IL-33/ST2 axis may help to restore an effective immune response. Immunotherapy strategies aimed at restoring immune function may also focus on blocking immune checkpoints (134) such as SIRPα. This protein, which inhibits phagocytosis through its interaction with CD47, is implicated in the disruption of phagocytic function in AMs. Thus, targeting SIRPα may prevent AMs paralysis (98).

### 5.2 Targeting microbiota to indirectly restore immune balance

With an increasing number of studies aiming to decipher the role of the microbiota in the onset of pulmonary infections, strategies based on microbiota modulation to indirectly enhance pulmonary immunity are emerging as promising therapeutic approaches (110, 135–137). As reviewed, infected lung display reduced abundance of genera belonging to the healthy lung core microbiome. In addition, murine models have demonstrated that host–microbiota crosstalk contributes to the maintenance of mucosal immunity. Therefore, restoring the initial diversity of the airway microbiome could help reestablish an effective host response. In a murine model, oral aspiration of three human oral anaerobic bacteria reduced susceptibility to subsequent *Streptococcus pneumoniae* pulmonary infection (138). Interestingly, two of these anaerobic bacteria have been identified as reduced in the microbiome of patients with hospital-acquired pneumonia (139). Rather than using whole bacteria, an alternative strategy involves identifying specific microbial components or conserved molecular patterns that modulate host immunity (140, 141). This approach relies on immunomodulation by targeting the crosstalk between commensal bacteria and immune cells, which can trigger protective effects against infection. For example, stimulation of Toll-like receptor (TLR) 5 (142) or TLR2 (121) has been associated with protection against *S. pneumoniae* infection. Similarly prophylactic treatment with TLR4 agonist was demonstrated to enhanced immune response to *K.pneumoniae* in mouse model (143). Of note, the use of TLR agonists in combination with antibiotic has been proposed to improve antibiotic efficacy (144). In mice, therapeutic synergy between antibiotics and TLR5 stimulation with FLAMOD has already been observed in both antibiotic-sensitive and -resistant pneumonia. A clinical study is currently underway to evaluate this drug in humans.

Modulation of the microbiota can also target symbiotic relationships between different microbial communities. One such strategy, inspired by gut microbiota modulation, is the modulation through microbial consortium (145). This approach focuses on identifying microorganisms that naturally cooperate symbiotically and can collectively influence immune processes. Microbial cooperation can also occur between different compartments. Since modifications of gut microbiota have been identified during pulmonary infections, recent studies have explored how interactions between the microbiota of both compartments may modulate each other. Targeting the gut–lung axis can be achieved through dietary interventions or ingestion of targeted substrates (prebiotics), as well as through the administration of one or two specific bacterial strains (probiotics). A clinical trial has already investigated the impact of a probiotic treatment composed of *L.rhamnosus* on the onset of ventilator-associated pneumonia, but failed to demonstrate a beneficial effect (146).

Despite the diversity of these approaches, including direct or indirect modulation of pulmonary environment, only a few clinical trials have been conducted. This is partly due to the difficulty to translate from experimental models to clinical.

### 5.3 Limitations of current evidence and research gaps

Indeed, immunomodulation in critically ill patients continues to face significant challenges in translation from bench to bedside. This is partly due to the lack of human-based data. Although pulmonary cells are relatively accessible, the invasive procedures required to obtain them, limit *ex vivo* research on human alveolar cells. While animal models have advanced our understanding of immunological alterations, particularly in organ-specific contexts, their translational relevance remains limited (147). These models are typically designed for homogeneity and reproducibility, which fails to capture the clinical heterogeneity of ICU patients, including variations in medical history and environmental exposures. Even with humanized animal models, inherent anatomical (148) and immunological (149) differences between species cannot be eliminated and must be carefully considered when extrapolating findings to humans. In the context of bacterial pneumonia, numerous murine models have been used to decipher the immune response. Still while neutrophils are central to the pulmonary defense, their phenotype, circulating levels, and immune-related protein expression differ markedly between mice and humans (150).

Beyond the limitations of animal models, research gaps in immunomodulatory strategies also stem from the difficulty of implementing experimental protocols in clinical settings. These challenges include technical constraints imposed by routine clinical practice and the high heterogeneity of patient profiles. Microbiota-targeted therapies exemplify these complexities (151). As previously discussed, microbiome analysis is technically complex and difficult to adapt to clinical workflow. For example, the core pulmonary microbiota includes anaerobic bacteria, which require oxygen-free conditions for sampling and storage. Moreover, the host–microbiome environment is highly dynamic and influenced by numerous confounding factors, complicating the identification of causal relationships or mechanisms underlying dysbiosis, an essential step in defining therapeutic targets. Longitudinal studies would help evaluate temporal dynamics but require repeated sampling, which is logistically challenging in critically ill patients.

Therapeutic strategies involving microorganisms face an additional major challenge in the context of personalized medicine: the high heterogeneity of patient profiles. Given our limited understanding of host–microbe interactions, microbiota modulation, particularly through the administration of whole microorganisms, while potentially beneficial, carries the risk of unintended and possibly harmful outcomes. This highlights both the need for more human data and for experimental approaches that are better aligned with clinical constraints.

## 6 Conclusion

Sepsis is a complex dysregulated host response to infection leading to organ dysfunction that may be heterogeneous and asynchronous at the organ level. This heterogeneity of the immune response may explain differences in susceptibility to secondary infections and mortality. In pneumonia-related sepsis, compartmentalisation of the immune response in the lung is associated with quantitative and phenotypic alterations of key partners such as AMs, polymorphonuclear cells and microbiota. Immune modulation with local delivery of cytokines such as IFN-γ or growth factors such as GM-CSF could help restore normal alveolar antibacterial defenses. In addition, modulation of the lung microbiota after an infection appears to be a promising way to improve the regional lung immune response to further infection.
